# Skin disease diagnosis using decision and feature level fusion of deep features

**DOI:** 10.3389/fdgth.2025.1478688

**Published:** 2025-10-17

**Authors:** Md. Zasim Uddin, Md. Arif Shahriar, Björn W. Schuller, Md. Nadim Mahamood, Md. Atiqur Rahman Ahad

**Affiliations:** ^1^ Department of Computer Science and Engineering, Begum Rokeya University, Rangpur, Bangladesh; ^2^ MRI, CHI—Chair of Health Informatics, Technische Universität München, Munich, Germany; ^3^ GLAM, Imperial College London, London, United Kingdom; ^4^ Department of Computer Science and Digital Technology, University of East London, London, United Kingdom

**Keywords:** skin disease diagnosis, deep learning, feature-level fusion, decision-level fusion, GAN, classification

## Abstract

**Introduction:**

Early skin disease diagnosis is essential and one of the challenging tasks for a dermatologist. Manual diagnosis by healthcare providers is subjective, costly, and may yield inconsistent results. In contrast, automated skin disease detection and classification using traditional machine learning and deep learning approaches have shown promise in addressing this problem.

**Methods:**

In this study, we propose a hybrid ensemble framework that integrates both feature-level fusion (FLF) and decision-level fusion (DLF) to leverage complementary strengths for detecting and classifying skin diseases. We employ two convolutional neural network (CNN)-based models, i.e., a modified DenseNet201 and VGG19, along with an attention-based model vision transformer (ViT) to identify and classify skin diseases. In FLF, feature representations from these models are point-wise added and passed through a shared classification head to make the final prediction. In DLF, decisions from each base model are collected, and the majority voting scheme is used to make a final decision. Furthermore, we incorporate a generative adversarial network (GAN)-based approach for offline-based training data augmentation to reduce overfitting and improve performance.

**Results:**

Based on different evaluation metrics (i.e., accuracy, precision, recall, and F1-score), our proposed framework demonstrates superior performance on four benchmark datasets: the PH2, HAM10000, ISIC 2018, and ISIC 2019 datasets, with an accuracy of 99.3%/99.2%, 92.7%/96.1%, 86.7%/89.0%, and 94.5%/95.0%, respectively, for FLF/DLF.

**Discussion:**

These results demonstrate that while both fusion strategies are effective, DLF slightly outperforms FLF, emphasizing the value of ensemble decision aggregation for robust skin disease classification.

## 1 Introduction

The human skin is the largest and most powerful organ in the body. It guards the body against outer temperature, ultraviolet rays, and harmful chemicals. Furthermore, the skin produces essential vitamin D in the human body. However, the human skin suffers from different causes, namely pollution, poor immunity, viruses, alcohol, unhealthy lifestyles, and ultraviolet light. Therefore, various diseases affect the human skin (1). Skin diseases are important public health problems that prevail in almost all age groups and are one of the most widespread kinds of illnesses worldwide (2). In the current context, diagnosing diseases still necessitates self-monitoring and regular medical examinations. In most cases, skin diseases can be tackled without any special treatment, whereas some of them lead to cancer and are life-threatening. The World Health Organization (WHO) reports that by the age of 70, one in five Americans will receive a diagnosis of skin cancer, with approximately 95,000 new cases being diagnosed daily in the US alone (3).

Early detection and treatment of skin disease are essential for reducing patient suffering and improving outcomes (4); otherwise, it may advance, possibly spread, and penetrate deeper layers of the skin, resulting in more severe stages of the condition (5). In extreme circumstances, skin diseases can lead to serious outcomes, including hindrance of daily functions, breakdown of relationships, and harm to internal organs, even death in cases like melanoma (a skin disease primarily characterized by the abnormal growth of melanocytes). Furthermore, they present a genuine risk of mental health issues such as isolation, depression, and potentially even suicide. However, if diagnosed early and properly treated, the survival rate can be as high as 97.0% (6).

For early diagnosis of skin disease, self-examination is a crucial step (7). The American Center for the Study of Dermatology developed an ABCD guideline so that individuals can be vigilant in recognizing asymmetry, wavy borders, color changes, and diameter on their skin (8). Later, manual diagnoses are employed to detect skin diseases by dermatologists or other healthcare providers. Dermoscopy is one of the very popular techniques (9) to detect skin disease by magnifying and lighting the skin surface and underlying structures (10). For further investigations, dermatologists may perform a skin biopsy for pathological examination if it is required (11). However, these types of manual diagnosis heavily rely on visual interpretation and subjective judgment. Particularly, clinicians with varying levels of experience, knowledge, and diagnostic abilities may obtain inconsistent diagnoses of skin diseases. Furthermore, it is costly and necessitates the use of specialized medical diagnostic tools such as dedicated laser-based devices, micro-spectroscopy, and other dermoscopy tools to locate the lesion (12).

To tackle this challenge and alleviate the burden of clinicians, automated computer vision and machine learning systems have been developed for computer-aided diagnosis (CAD) systems for skin disease detection and diagnosis (13, 14). The use of CAD is convenient, less expensive, and faster (15), and systems can be divided into two categories: traditional machine learning (ML) and modern deep learning (DL)-based methods. Traditional machine learning (ML) approaches rely on manually hand-crafted features, typically involving pre-processing and extracting features like texture, color, size, and shape, followed by classification using methods such as gradient boosting, SVM, or artificial neural networks (ANN). Different ML-based approaches were employed for skin disease diagnosis in the literature; for example, Ahammed et al. (16) utilized Decision Tree (DT), Support Vector Machine (SVM), and K-Nearest Neighbor (KNN) models for skin disease detection and classification. Similarly, Jagdish et al. (17) employed KNN and SVM with wavelet analysis for skin disease detection and classification. However, applying these traditional ML methods to new, unfamiliar scenarios is often challenging.

In contrast, DL-based methods are convenient as they can automatically extract features and reduce errors, leading to better performance (18). They have produced promising results for the detection and classification of skin disease (19–22). For example, Abd et al. (22) developed a robust DL-based model for the classification of skin disease that uses MobileNetV3 for features extraction purposes. Khan et al. (20) used deep convolutional neural network-based models such as VGG and AlexNet to classify skin disease. Similarly, Brinker et al. (19) used the residual network ResNet50 for skin disease classification. Most studies rely on a single end-to-end model, and such models are prone to overfitting and hinder the adaptability and generalizability to other unfamiliar datasets.

To overcome these limitations, we propose a DL-based ensemble framework that classifies skin disease using feature-level fusion (FLF) in an end-to-end way and fusion at the decision level for a non-end-to-end manner for decision-level fusion (DLF). FLF merges feature representations before classification, allowing the model to learn richer, more fine-grained complementary information of lesions in a shared space, whereas DLF aggregates final predictions from multiple base models, reducing bias from a single base model. Using both allows the system to benefit from joint representation learning for FLF while still leveraging the robustness of majority voting for the DLF. More specifically, we demonstrated that DLF slightly outperforms FLF on most benchmarks, but the combination offers insights into which level of fusion is more beneficial for specific datasets. The contribution of this study is summarized as follows:

• We introduce a comprehensive end-to-end ensemble framework for diagnosing skin diseases, comprising two CNN-based and an attention-based vision transformer model. The features extracted from these base models are fused at a feature level to generate conclusive features in the final layer and employ Softmax for diagnosis. In addition, the individual classifiers’ decisions are merged using a majority voting technique to make the final decision for the skin disease diagnosis.
• We utilize data augmentation with a deep generative adversarial network (GAN) to produce additional training data. Through empirical investigations on the benchmark datasets, we observe a notable improvement in the performance using data augmentation.
• We evaluate the proposed framework on four publicly available skin disease datasets: PH2, HAM10000, ISIC 2018, and ISIC 2019. The results demonstrate that the proposed framework achieves superior performance compared to various metrics such as accuracy, precision, recall, and F1-score.

## 2 Related work

### 2.1 CNN-based approaches

Convolutional neural networks (CNNs) have been remarkably efficient methods for handling pre-processing, extracting features, and performing classification in various domains of computer vision, including biometrics (23, 24), medical imaging, as well as diagnosis of skin diseases (25–29). Some studies (25, 26) propose dedicated CNN architectures for skin disease classification. For example, Shanthi et al. (25) implemented an architecture consisting of 11 layers, incorporating convolution, pooling, fully connected (FC) layers, and Softmax for classification. On the other hand, four convolutional layers, two max-pooling layers, one FC layer, and three dense layers are found in (26). By contrast, some studies (27, 30, 31) employed existing pre-trained models for the classification of skin disease. For example, Muhaba et al. (27) utilized a pre-trained MobileNet CNN model and demonstrated it on a dataset collected from a clinic using different smartphone cameras. In contrast, the studies in (32) used four different CNN-based models: DenseNet121, ResNet50, VGG16, and ResNet18, and demonstrated on the HAM10000 dataset and found out that the ResNet50 obtained the best accuracy at 90.0%. Furthermore, Kousis et al. (30) conducted a study on the identification of skin lesions using 11 different CNN architectures. They demonstrated the classification of seven different types of skin lesions, where the DenseNet169 model achieved the best performance at 92.2%, 93.6%, and 93.3%, of accuracy, sensitivity, and F1-score, respectively, compared to the other end-to-end CNN architecture using the HAM10000 dataset. Similarly, Mondal et al. (31) utilized a modified-DenseNet201 by replacing the last layers with a single global average pooling layer, five FC layers, dropout, and finally, one Softmax layer for classification and showed that it outperforms the existing DenseNet169 and DenseNet121 models where it gains 13.8% more accuracy than the non-modified DenseNet201 on the HAM10000 dataset. Similarly, Karthik et al. (33) have proposed a modification to the EfficientNet V2 model for the classification of skin disease. Specifically, they replaced the standard Squeeze-and-Excite block with an Efficient Channel Attention block. Shan et al. (34) introduced a convolutional Block Attention Module (CBAM) and used it in combination with DenseNet121 to enhance the feature representation capabilities. Additionally, they utilized an improved focal loss algorithm to deal with data imbalance effectively. These modifications have shown promising results in improving the performance of the model and achieving an AUC of 0.99 on the HAM10000 dataset. Similarly, Raghavendra et al. (35) used a model with CNN and a global average pooling layer to classify skin diseases. They also implemented the black hat filtering approach and the resampling technique to remove artifacts and increase data, which aided in outperformance by achieving accuracy at 97.2% on the HAM10000 dataset.

In addition, several studies explored the uses of CNN-based models for feature extraction. For example, the studies in (22, 36) implement a lightweight MobileNet for feature extraction. Additionally, the authors used Long Short-term Memory (LSTM) in (36) and the Artificial Rabbits Optimizer in (22) and achieved an accuracy at 87.2%, 96.8%, and 88.7% on the ISIC 2016, PH2, and HAM10000 datasets, respectively, while an accuracy of 85.3% was reached in (36) on the HAM10000 dataset. Similarly, Yu et al. (37) employed ResNet50 to extract features and obtain the global feature descriptor using a fisher vector and finally classified skin diseases using SVM with a Chi-squared kernel. They validated their model on the ISBI 2016 challenge dataset, achieving accuracy and AUC at 86.8% and 85.2%, respectively. Similarly, Hameed et al. (29) utilized AlexNet for feature extraction and an SVM for classification. They evaluated a privately collected dataset and discovered that their approach achieved an accuracy of 86.2%. On the other hand, Seeja et al. (38) used U-Net in conjunction with an SVM for classification, demonstrating its effectiveness on the ISBI 2016 dataset. Their approach achieved an accuracy of 85.2%, precision of 42.6%, recall of 50.0%, and F1-score of 46.0%. In contrast, Bandyopadhyay et al. (39) employed AlexNet, GoogLeNet, ResNet50, and VGG16 for feature extraction, and used SVM, AdaBoost, and Decision Tree classifiers for classification using the ISIC 2016 challenge dataset.

Some studies employed segmentation techniques to segment the area of the disease lesion, and subsequently, lesions were utilized to enhance the classification accuracy. Son et al. (40) proposed a two-stage approach to classify skin diseases. In the first stage, they implement a U-net architecture to decompose and normalize the input images, generating a segmentation map of the skin lesion. In the second stage, they introduce EfficientNets to classify the segmented images. This approach showed promising results in accurately identifying various skin diseases. Similarly, Adla et al. (41) utilized Tsallis entropy-based segmentation to detect the lesion area. Later, the classification of segmented lesions was done using a convolutional sparse Autoencoder. Furthermore, Kalpana et al. (28) segmented the malignant lesion using a threshold-based technique and classified it through an ensemble model with an SVM classifier and a random forest kernel. In addition, Zhu et al. (42) employed a CNN-based model for both binary classification (i.e., benign vs. malignant) and multiclass classification using high-frequency ultrasound images of skin lesions.

All of the single end-to-end or custom CNN-based models used a traditional convolutional approach, which may similarly extract the features, leading to robustness on a single dataset and less generalize on other datasets (43). However, end-to-end methods are necessary for real-world applications because they can automatically extract relevant features directly from raw data, reduce multiple processing stages, and make decisions based on the features, which is particularly necessary where manual feature extraction is challenging, for example, skin disease detection and identification. In this study, we propose an ensemble framework and perform experiments end-to-end as a feature-level fusion and a decision-level fusion.

### 2.2 Vision transformer-based approaches

The Vision Transformer (ViT) (44)-based approach represents attention-based architectures showcasing the effectiveness of attention mechanisms in capturing extensive spatial relationships within images. These models partition an image into non-overlapping patches of fixed size, subsequently transforming them into a sequence of vectors through linear embedding. Similar to CNN-based approaches, ViT models are widely used for segmentation (45), detection and classification (46), as well as for skin disease diagnosis and classification (12, 47, 48). For example, Aladhadh et al. (12) employed a ViT model along with data augmentation for skin cancer diagnosis. They demonstrated on the HAM10000 dataset and found that the ViT-based model obtained better accuracy than CNN-based approaches for the classification of skin cancer with accuracy, precision, sensitivity, and F1-score at 96.1%, 96.0%, 96.5%, and 97.0%, respectively. Similarly, Xin et al. (47) introduced a framework including a multi-scale vision transformer and multi-scale patch embedding technique to improve the image features and finally apply contrastive learning for skin disease classification. Their proposed approach obtained accuracy, precision, and AUC at 94.3%, 94.1%, and 98.0%, respectively, on the HAM10000 dataset. Further, Nie et al. (49) employed a two-stage model including a CNN-based module to extract local and low-level features, a ViT model for the high-level semantic information from these features, and finally, a multi-layer perceptron (MLP) head was used for the classification of skin disease, and achieved accuracy, precision, recall, and F1-score at 89.5%, 89.6%, 89.5%, and 89.1%, respectively, on the HAM10000 dataset. In addition, Dai et al. (48) introduced the HierAttn model, which uses a multi-stage and multi-branch attention mechanism to simultaneously learn local and global contextual features while maintaining a lightweight architecture. This is particularly suitable for real-time and mobile-based applications in skin disease diagnosis, and classification.

### 2.3 Fusion-based approaches

Feature-level fusion (FLF) and decision-level fusion (DLF) are the most commonly used techniques for ensemble learning for skin disease diagnosis. In FLF, concatenation or pointwise addition of the extracted features from the multiple base models takes place. In contrast, in DLF, the decision of the base classifiers is averaged or selected by majority voting for the final decision. Regarding FLF, Wang et al. (50) introduced a multiscale feature fusion model for classifying skin disease using DenseNet121 and an improved VGG16. They demonstrated its performance on the HAM10000 dataset, achieving an accuracy of 91.2%, while Gairola et al. (51) introduced a multi-feature fusion approach using different deep networks to improve accuracy. Similarly, Elashiri et al. (52) extracted features from ResNet50, VGG16, and Deeplabv3 and concatenated them at the feature level. These concatenated features were sent to the feature transformation stage for weighted feature extraction, and finally, LSTM was employed for classification. They evaluated the PH2 and HAM10000 datasets and obtained an accuracy of 93.5% and 93.8%, respectively, for the PH2 and HAM10000 datasets. Similarly, Afza et al. (53) introduce an approach including image acquisition and enhanced contrast, feature extraction using deep learning, and selecting the best feature using entropy-mutual information and fuse by employing a modified canonical correlation. They evaluated the HAM10000 and ISIC2018 datasets and found that their framework achieved an accuracy of 93.4% on both datasets.

In contrast, Dang et al. (54) proposed an ensemble model comprised of five CNN-based models: Inception-v3, Densenet169, ResNet50, Inception-ResNet-v2, and Xception, along with Squeeze-and-Excitation Blocks to emphasize on informative features. They employed majority voting for decision-level fusion. They obtained accuracy, precision, recall, F1-score, and AUC at 90.9%, 85.9%, 80.8%, 82.8%, and 91.1%, respectively, on the ISIC 2017 dataset. Similarly, Harangi (55) proposed an ensemble model where they considered four CNN-based methods: VGG, ResNet, GoogLeNet, and AlexNet. They employed the weighted average technique for the final prediction of the skin disease. They achieved an AUC of 0.891 on the official test dataset of the IEEE International Symposium on Biomedical Imaging (ISBI) 2017 challenge on Skin Lesion Analysis Towards Melanoma Detection. We observed that most of the methods employed either FLF or DLF; however, in this study, we studied extensively FLF and DLF in our ensemble framework.

## 3 Methodology

### 3.1 Overview

**Figure 1:**
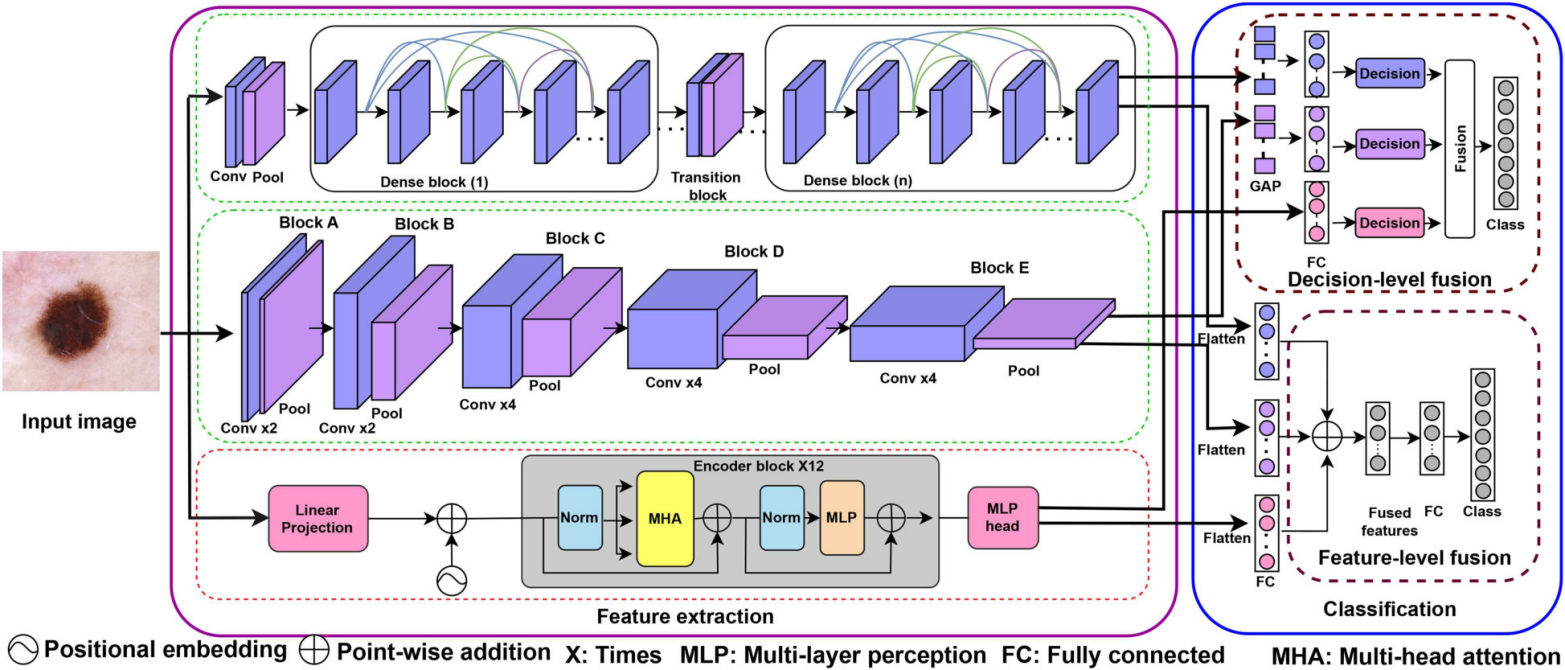
Overview of our proposed ensemble framework for the feature extraction (left side) and classification (right side) of skin disease. For feature extraction, the framework includes three modules: two CNN-based models and an attention-based model. For classification, the framework includes feature-level fusion (FLF) and decision-level fusion (DLF). The extracted features from base models are fused as pointwise addition for FLF, while majority voting techniques are employed for DLF.

In this study, we propose a novel ensemble framework that leverages the complementary strengths of three modules to extract smart features: two of which are CNN-based, and the other is an attention-based Vision Transformer (ViT). An overview of the proposed framework is presented in Figure 1. Our framework is based on a modified DenseNet201 (56), and VGG19 (57) as a CNN-based approach, while Vision Transformer (ViT) (44) is an attention-based vision transformer model. The fused features are subsequently fed into a fully connected embedding layer. Finally, a single-layer classification network with a Softmax activation function is employed. This network calculates the cross-entropy loss for end-to-end classification, realizing feature-level fusion (FLF). Additionally, the decision of each individual model is employed to fuse for the final decision for decision-level fusion (DLF) as a majority voting technique.

#### 3.1.1 CNN-based model

Our CNN-based approaches are based on the modified DenseNet201 and VGG19 architecture.

*DenseNet* (56) is a high parametric efficient CNN-based model. It reuses the features from different layers, which increases the variety of input for subsequent layers. Additionally, it prevents vanishing gradients by dense connections between layers and also ensures no loss of information (58) and efficient memory consumption (59). DenseNet has different versions, which are categorized based on the number of layers. In our proposed framework, we exploit the DenseNet201, which consists of 201 layers. The fundamental component of DenseNet is a defined number of dense blocks along with a transition block. At first, an input image X with spatial resolution H×W, where H, and W stand for height and width, respectively, are passed through a 7×7 convolution and 3×3 max-pooling layers and produces an output feature map $Z_{0}^{0}$ with dimension $M_{0}^{0}\times N_{0}^{0}$, and can be expressed as:

$$Z_{0}^{0}=M\_Pool(Conv(X)),$$

where, Conv(⋅) is convolution, while M_Pool(⋅) stands for max-pooling. Then, the feature map passes through several dense blocks and transition layers. In a dense block, each layer takes input from all preceding layers. Each dense block begins with a bottleneck layer, a 1×1 convolution layer, which decreases the number of channels in the input feature maps, followed by a 3×3 convolution layer that is densely interconnected. For the kth block, it can be expressed as follows:

$$Z_{k}^{l}=H_{k}(cat[Z_{k}^{0},Z_{k}^{1},\ldots ,Z_{k}^{l-1}]),$$

where, $H_{k}(\cdot )$ is a non-linear transformation that comprises batch normalization, ReLU, and convolution, and generates a feature map $Z_{k}^{l}$ with dimension $M_{k}^{l}\times N_{k}^{l}$ in the lth layer along with the kth dense block, while cat(⋅) is the concatenation of all preceding layers’ feature map $Z^{0},Z^{1},\ldots ,Z^{l-1}$, respectively, for the layers 0,1,…,l−1.

Furthermore, a transitional block is introduced between dense blocks to reduce the size of the feature maps and the number of channels. The transition includes a BN layer, a 1×1 convolutional layer, and an average pooling layer with a stride of 2×2. Later, an FC layer with dimension $De_{d}$ is added to extract features and fuse with other features [in Equation 1] for an end-to-end feature-level fusion (FLF). Regarding the decision-level fusion (DLF), a global average pooling (GAP) layer is exploited to aggregate the spatial information into a fixed-length feature vector and Softmax layer classification.

*VGG19* (57) is the most widely explored method for image classification. A series of stacked convolutional layers are the foundation of the VGG19 structure, which is then followed by FC layers. The convolutional part is made up of 16 convolutional and is divided into five blocks and three FC layers with ReLU activation. Each convolution consists of a 3×3 kernel with a 2×2 pooling layer. Firstly, the input image, X, is passed through Block A consists of two consecutive convolutions and max-pooling along with 64 number of channel and generates a feature map, $X_{i1}$ with dimension $K_{2}\times L_{2}$ and is outlined as:

$$X_{i1}=M\_Pool(Conv(Conv(X))),$$

where, Conv(⋅), and M_Pool represents convolution and max-pooling respectively. Afterward, this feature map is passed through Block B in the same way as Block A, generating a feature map $X_{i2}$, and sent to the Block C consisting of four consecutive convolutions followed by max-pooling along with 256 channels, generates a feature map $X_{i3}$ with a size of $K_{4}\times L_{4}$. Similarly, the feature map $X_{i3}$ is sent to Block D with four consecutive convolutions followed by max-pooling along with 512 channels, and generates a feature map $X_{i4}$, then this is sent to Block E and generates the final feature map $X_{i5}$, using in the same way as Block D. For more information, please follow the original paper (57).

Finally, we added an FC layer after the Block E to have the same dimensions as the $Vg_{d}$, which encodes rich spatial information and fuses with the FLF used in Equation 1. By contrast, we employed a global average pooling (GAP) layer to aggregate the spatial information into a fixed-length feature vector and Softmax layer classification for the DLF, as shown in Figure 1.

#### 3.1.2 Attention-based model

Our framework employs a Vision Transformer-based model (44), which applies the standard multi-head self-attention (MHSA) mechanism originally introduced for natural language processing (60). The input image $X\in R^{H\times W\times C}$ is reshaped into $N=HW/P^{2}$ non-overlapping patches of size P×P, linearly projected to a latent dimension D, and prepended with a learnable class label $X_{\mathrm{cls}}$. A positional embedding $E_{\;pos}$ is added to preserve spatial relationships, it can be represented as:

$$Z_{0}=\left[X_{\mathrm{cls}};X_{\mathrm{pch}}^{1}E;\ldots ;X_{\mathrm{pch}}^{N}E\right]+E_{\mathrm{pos}}.$$

The resulting sequence is passed through several encoder layers, each containing a standard MHSA block followed by a two-layer feed-forward multi-layer perceptron (MLP) with GELU activation and residual connections. The MHSA computes attention as:

$$A(Q,K,V)=\mathrm{Softmax}\left(\frac{QK^{T}}{\sqrt{D}}\right)V,$$

where Q, K, and V are the learned query, key, and value projections. The outputs from all heads are concatenated and linearly projected to produce the final representation. For additional information on the MHSA formulation, see (44). Finally, the $Vi_{d}$ features from the MLP head are used for FLF in Equation 1, while the classification decision is used for DLF.

### 3.2 Feature-level fusion (FLF)

We employ the point-wise addition of the extracted features (e.g., $De_{d}$, $Vg_{d}$, and $Vi_{d}$) from the previously mentioned base models in our proposed ensemble model for feature-level fusion, which can be performed as follows:

(1) $$F_{\mathrm{fused}}=P_{\mathrm{add}}([De_{d},Vg_{d},Vi_{d}]),$$

where $P_{\mathrm{add}}(\cdot )$ denotes point-wise addition of the feature vectors. Prior to fusion, each feature vector is normalized to ensure comparable scale and distribution across the CNN and ViT models. After that, we employ a FC layer with 512 dimensions to enable the model to learn appropriate weighting and alignment of the fused features during training. Finally, a Softmax layer produces the output probabilities for skin disease classification in an end-to-end manner:

$$Y_{\mathrm{class}}=\mathrm{Softmax}(\mathrm{FC}(F_{\mathrm{fused}})).$$

### 3.3 Decision-level fusion (DLF)

Decision-level Fusion (DLF) combines the decisions for each of the classifier’s decisions instead of only a single model. In our framework, we consider the majority voting strategy to count the votes received from each classifier. The class with the most votes is chosen as the consensus decision, and the overall procedure can be outlined as follows:

$$\begin{array}{rl} P_{A} & =De_{A}(X)\\ P_{B} & =De_{B}(X)\\ P_{C} & =De_{C}(X)\\ Y_{\mathrm{class}} & =MV(P_{A},P_{B},P_{C}), \end{array}$$

where, X represents the input image, and $P_{A}$, $P_{B}$, and $P_{C}$ represent the prediction classes using the modules A, B, and C, respectively, while MV(⋅) denotes majority voting. The overview for the DLF portion is shown in Figure 1.

## 4 Datasets and evaluation metrics

To demonstrate the proposed framework for diagnosing skin diseases, experiments were conducted on four publicly available benchmark datasets.

### 4.1 Datasets

*PH2 dataset* (61) is a dataset with three skin disease classes: Atypical Nevus (AN), Common Nevus (CN), and Melanoma (MEL) captured from the Dermatology Service of the Hospital Pedro Hispano, Matosinhos, Portugal. It comprises 200 images that were captured under identical conditions and instrumentation resolution. We followed the K-fold cross-validation technique to ensure a robust and unbiased evaluation of our proposed method. Specifically, we used K = 5, dividing the dataset into five equal parts. In each iteration, four folds were used for training and the remaining one for testing, with the test fold rotating across the five runs. Finally, the results were averaged across all five folds. The benchmark dataset used in this evaluation is denoted as PH2 in the experimental discussions.

**Figure 2:**
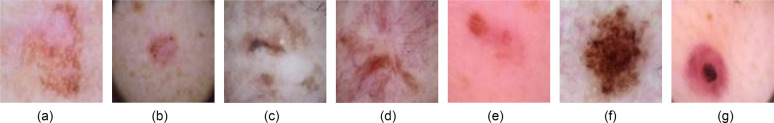
Example images for each skin disease from the HAM10000 dataset, where AKIEC, actinic keratosis; BCC, basal cell carcinoma; BKL, benign keratosis; DF, dermatofibroma; NV, melanocytic nevi; MEL, melanoma; VASC, vascular lesions. **(a)** AKIEC. **(b)** BCC. **(c)** BKL. **(d)** DF. **(e)** NV. **(f)** MEL. **(g)** VASC.

*HAM10000* (62) is a training subset of the ISIC 2018 challenge dataset, including 10,015 training dermatoscopic image samples. The dataset includes images of seven types of skin disease: Actinic keratosis (AKIEC), Basal cell carcinoma (BCC), Benign keratosis (BKL), Dermatofibroma (DF), Melanocytic nevi (NV), Melanoma (MEL), and Vascular lesions (VASC). The data were captured over 20 years from Australia and Austria from 54.0% male and 45.0% female participants. An example sample image for each class is shown in Figure 2. Initially, the HAM10000 dataset was released only as a training set, with no corresponding official test labels provided. Consequently, numerous state-of-the-art studies adopted a common practice of splitting the HAM10000 dataset (i.e., 80:20 ratio) into training and testing subsets for performance evaluation. Following this widely used approach, we similarly conducted experiments for a fair comparison with prior works. The benchmark dataset is denoted by HAM10000 in the experiment discussions.

*ISIC 2018*^1^ is an official test dataset released by the ISIC 2018 challenge organizers, consisting of 1,512 dermatoscopic images covering the same seven classes: AKIEC, BCC, BKL, DF, NV, MEL, and VASC. Unlike HAM10000, which was originally provided solely as a training set, the ISIC 2018 dataset includes ground-truth labels for the test samples, enabling independent evaluation of model performance. Following the official ISIC 2018 challenge protocol, the HAM10000 dataset is used for training, and the ISIC 2018 dataset serves as the independent test set. This setup provides a robust assessment of the model’s generalizability to unseen data beyond the HAM10000 distribution. The benchmark dataset is denoted by ISIC 2018 in the experiment discussions.

*ISIC 2019*^2^ is a further challenge training dataset that comprises two datasets, namely HAM10000 and BCN_20000. It includes a total of 25,331 images. The dataset covers eight different skin disease categories, which are AKIEC, BCC, BKL, DF, MEL, NV, Squamous cell carcinoma (SCC), and VASC. For a fair comparison with the existing approaches, we followed the same protocol as the dataset was randomly divided into 90% for training and the remaining 10% for testing.

### 4.2 Data augmentation

The deep learning-based approaches require large-scale training data to enhance performance and mitigate the risk of overfitting. A common strategy to address this challenge involves artificially augmenting the training samples to allow the models to gain a deeper understanding and insight. Typically, there are two types of data augmentation—offline and online data augmentation—for computer vision (63, 64). Pre-training data augmentation involves the a priori application of image transformations to the training set. This process generates augmented images, which are then stored alongside their original counterparts within the dataset. During model training, both the original and augmented data are utilized. In contrast, real-time data augmentation entails the application of image transformations on a per-batch basis during the training process. These transformations effectively generate variations of the original training images, which are subsequently fed into the model for training. Common real-time augmentation techniques encompass rotation, resizing, horizontal and vertical flipping, and cropping.

**Figure 3:**
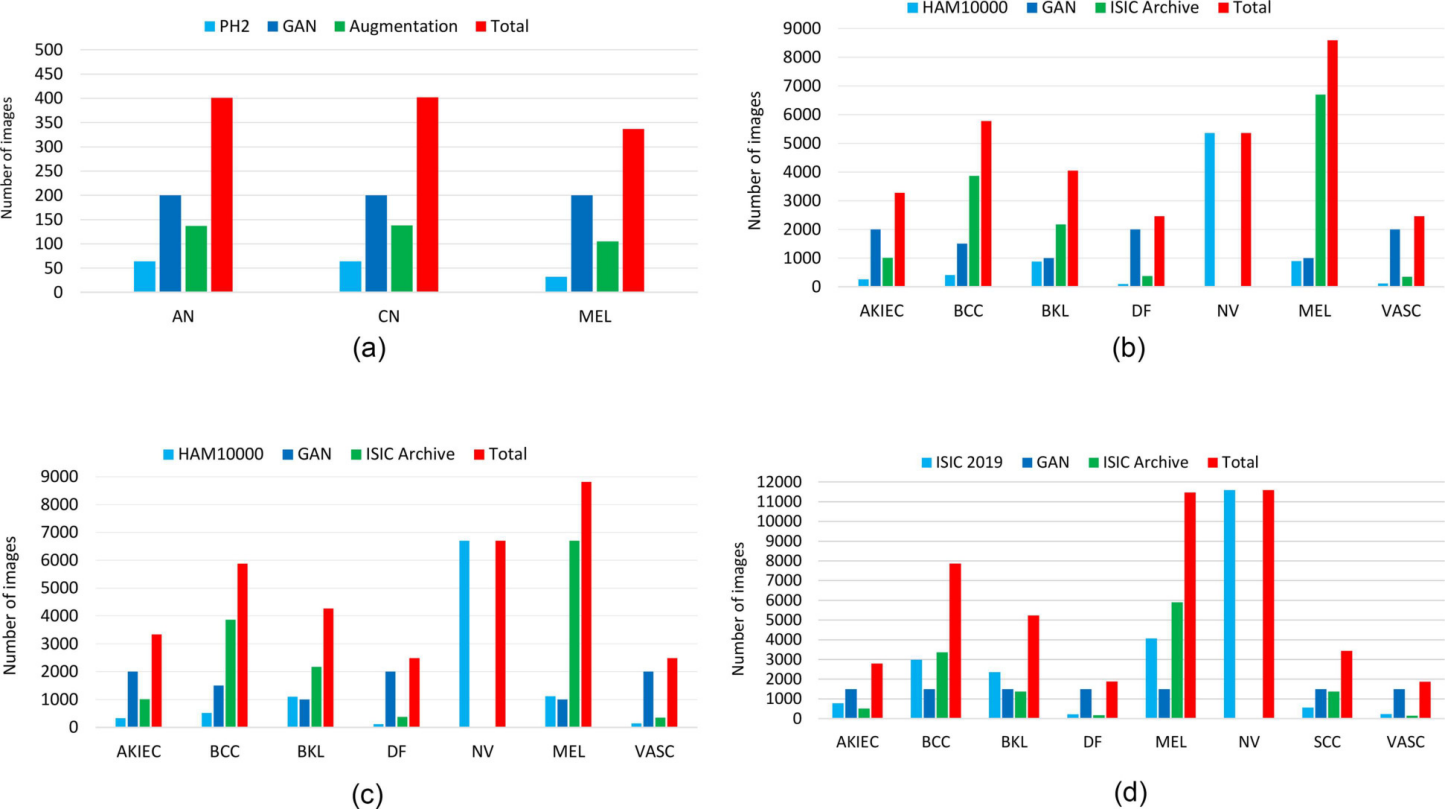
The distribution of the training samples for each class in the different datasets before and after data augmentation, using a generative adversarial network (GAN) and with ISIC archives. **(a)** PH2. **(b)** HAM10000. **(c)** ISIC 2018. **(d)** ISIC 2019.

To augment the training set, we employ a pre-training strategy that leverages a generative adversarial network (GAN) (65) for data augmentation. Additionally, we incorporate images from the ISIC archive^3^ to increase the training data volume. Furthermore, we employ rotation, resizing, and cropping as online data augmentation. The class-wise distribution of the sample skin disease images before and after augmentation is presented in Figure 3.

**Figure 4:**
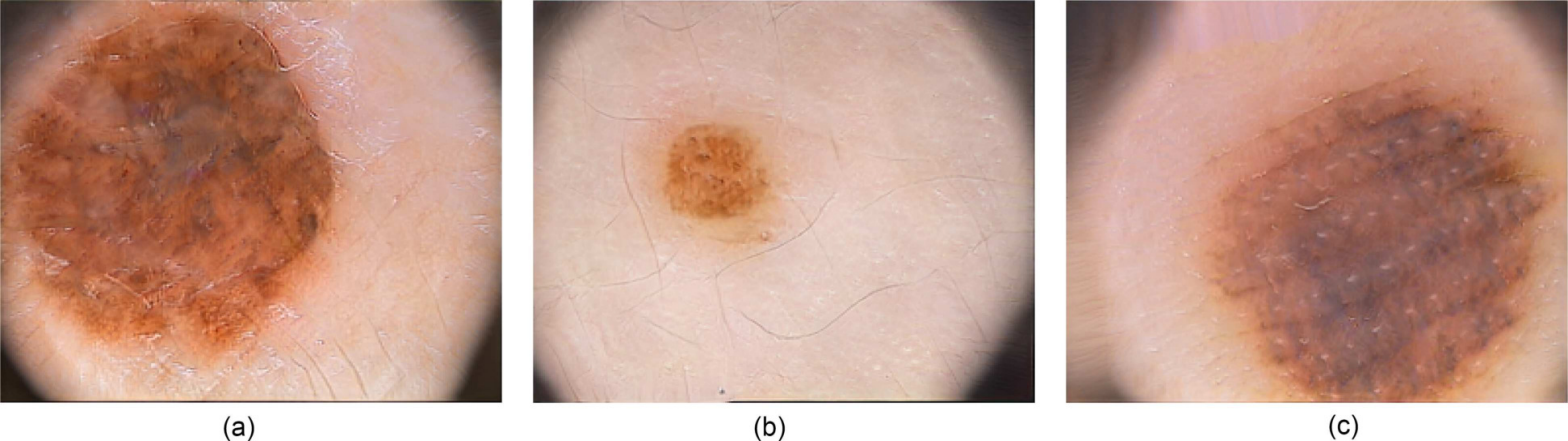
Example of images generated by the generative adversarial network (GAN) for the PH2 dataset. **(a)** Atypical Nevus (AN). **(b)** Common Nevus (CN). **(c)** Melanoma (MEL).

**Figure 5:**
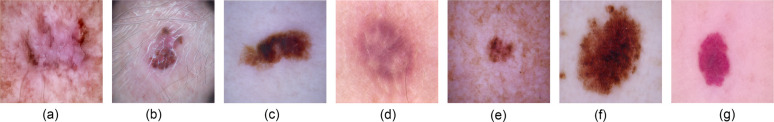
Example of images generated by the generative adversarial network (GAN) for the HAM10000 dataset, where AKIEC, actinic keratosis; BCC, basal cell carcinoma; BKL, benign keratosis; DF, Dermatofibroma; NV, Melanocytic nevi; MEL, Melanoma; VASC, Vascular lesions. **(a)** AKIEC. **(b)** BCC. **(c)** BKL. **(d)** DF. **(e)** NV. **(f)** MEL. **(g)** VASC.

To assess the quality of the GAN-generated sample images, we computed the Fréchet Inception Distance (FID) (66) scores across datasets and classes. The generated images achieved average FID scores of 82.9 for PH2, 37.8 for HAM10000, and 36.5 for ISIC 2019. Lower FID values indicate a higher similarity between the generated and real images, suggesting that the generated samples are visually realistic and diverse overall. The detailed class-wise FID scores are summarized in Table 1, and representative examples of the generated images are shown in Figures 4, 5 for the PH2 and HAM10000 datasets, respectively.

**Table 1:** Fréchet Inception Distance (FID) scores for sample images generated by the generative adversarial network (GAN) across datasets and disease classes.

Disease name	PH2	HAM10000	ISIC 2018	ISIC 2019
AKIEC	–	40.5	40.5	38.8
BCC	–	38.3	38.3	36.9
BKL	–	29.0	29.0	27.8
DF	–	40.7	40.7	39.1
NV	–	29.4	29.4	27.2
VASC	–	50.5	50.5	44.1
SCC	–	–	–	42.4
AN	80.6	–	–	–
CN	83.2	–	–	–
MEL	84.8	35.0	35.0	35.4
**Average**	**82.9**	**37.8**	**37.8**	**36.5**

A lower FID score indicates higher similarity to real images. A “–” denotes the absence of a particular disease class in the respective dataset.

### 4.3 Evaluation metrics

We evaluate the effectiveness of our proposed framework using different evaluation criteria: Accuracy, Precision, Recall, F1-score, Balanced accuracy, ROC (Receiver Operating Characteristic), and AUC (Area Under the Curve) (67). These evaluation metrics are calculated from the confusion matrices’ key four parameters, i.e., True Positives (TP), True Negatives (TN), False Negatives (FN), and False Positives (FP). TP refers to the number of instances correctly predicted as a positive class, and TN refers to the number of instances correctly predicted by the model as belonging to the negative class. On the other hand, FP is the number of instances where the model incorrectly predicts the positive class, and FN is the number of instances where the model incorrectly predicts the negative class.

In addition, we consider ROC, which visualizes the trade-off between True Positive Rate (TPR) and False Positive Rate (FPR) across classification thresholds, while AUC quantifies the model’s overall performance, with higher values indicating better discrimination between classes, where 1.0 represents perfect classification and 0.5 indicates random guessing. *Accuracy* is the ratio of correct predictions made by the model out of the total number of predictions, and can be calculated as follows:

$$Accuracy=\frac{\left|\mathrm{TP}+\mathrm{TN}\right|}{\left|\mathrm{TP}+\mathrm{TN}+\mathrm{FP}+\mathrm{FN}\right|}$$

*Precision* measures the proportion of true positive predictions out of all positive predictions made by the model and is calculated as:

$$Precision=\frac{\left|\mathrm{TP}\right|}{\left|\mathrm{TP}+\mathrm{FP}\right|}$$

*Recall* also known as True Positive Rate, TPR measures the proportion of true positive predictions out of all actual positive instances in the experiment, which can be calculated as:

$$Recall=\mathrm{TPR}=\frac{\left|\mathrm{TP}\right|}{\left|\mathrm{TP}+\mathrm{FN}\right|}$$

*False Positive Rate* (FPR) measures the proportion of false positive predictions out of all actual negative instances, calculated as:

$$FPR=\frac{\left|\mathrm{FP}\right|}{\left|\mathrm{FP}+\mathrm{TN}\right|}$$

*F1-score* is the harmonic mean of precision and recall; it strikes a balance between precision and recall, making it an effective metric for assessing both false positives and false negatives. The F1-score is calculated as follows:

$$F1-score=\frac{2(\mathrm{Precision})(\mathrm{Recall})}{\mathrm{Precision}+\mathrm{Recall}}$$

*Balanced accuracy (BACC)* is an evaluation metric used to evaluate the accuracy of a classification model when dealing with imbalanced datasets. It is defined as the average recall for each class.

$$BACC=\frac{1}{N_{\mathrm{classes}}}\sum_{i}^{N_{\mathrm{classes}}}{\mathrm{Recall}}_{i}$$

## 5 Experiments and results

In this section, we will introduce the system implementation and performance of the proposed framework.

### 5.1 Implementation details

The proposed framework was implemented by leveraging the TensorFlow library on an NVIDIA GeForce RTX 3090 GPU. The AdamW optimizer with a learning rate of $1\times e^{-4}$, a weight decay of $4\times e^{-3}$ and an epsilon of $1\times e^{-7}$ were used to optimize our proposed framework. Additionally, categorical cross-entropy loss is used as a loss function. We employed 150 epochs with 8 mini-batch sizes to train our end-to-end FLF and DLF framework. The learning rate (LR) was reset to 1e-5 after 50 epochs and again reset to 1e-6 after 100 epochs. Moreover, the the dimension d was set to 768 for the $De_{d}$, $Vg_{d}$, and $Vi_{d}$ in Equation 1.

### 5.2 Comparison with SOTA methods

#### 5.2.1 Evaluation of PH2 dataset

The accuracy, precision, recall, and F1-score on the PH2 dataset are presented in Table 2 and Supplementary Figure S1. Compared with the CNN-based method for feature extraction and then feeding these features into the ML-based classifier (68, 70, 72), the custom CNN-based model (71), and CNN-based End-to-End models (68, 69), our proposed feature-level fusion (FLF) and decision-level fusion (DLF) ensemble framework achieved an accuracy of 99.3%, and 99.2% respectively for the FLF and DLF. Moreover, we observe that the proposed FLF approach achieves performance comparable to the best existing method reported by Maniraj et al. (69), while also providing consistently high precision, recall, and F1-scores.

**Table 2:** Comparison of the proposed framework with existing methods applied to the PH2 dataset.

Reference	Method	Accuracy	Precision	Recall	F1-score
Benyahia et al. (68)	DenseNet+SVM	99.0	–	–	–
Maniraj et al. (69)	VGG	**99.3**	*99.2*	**99.4**	–
Elashiri et al. (52)	ResNet50+VGG16+DeepLabv3	93.5	90.4	–	–
Afza et al. (70)	ResNet50+NB	95.4	95.3	–	95.2
Reddy et al. (71)	CNN	94.2	96.2	91.8	93.9
Maqsood et al. (72)	Xception+ResNet50 ResNet101+VGG16+SVM	98.9	–	–	–
Mustafa et al. (73)	ResUNet+AlexNet	94.2	–	–	–
Our	FLF (DenseNet201+VGG19+ViT)	**99.3**	**99.3**	*99.3*	**99.3**
Our	DLF (DenseNet201+VGG19+ViT)	*99.2*	*99.2*	99.2	99.2

Bold values indicate the best benchmark.

#### 5.2.2 Evaluation of the HAM10000 dataset

**Figure 6:**
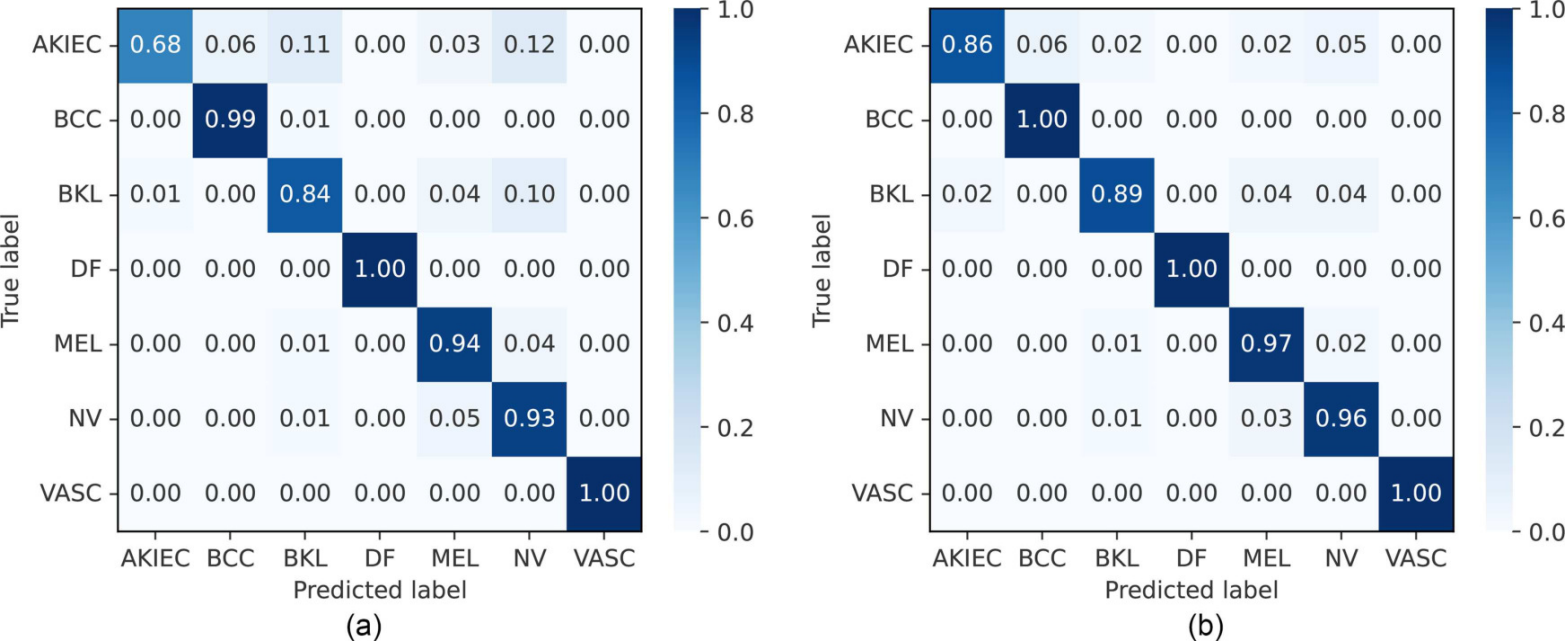
Normalized confusion matrices of FLF and DLF approaches on the HAM10000 dataset. AKIEC, actinic keratosis; BCC, basal cell carcinoma; BKL, benign keratosis; DF, dermatofibroma; NV, melanocytic nevi; MEL, melanoma; VASC, vascular lesions. **(a)** Feature-level fusion. **(b)** Decision-level fusion.

**Figure 7:**
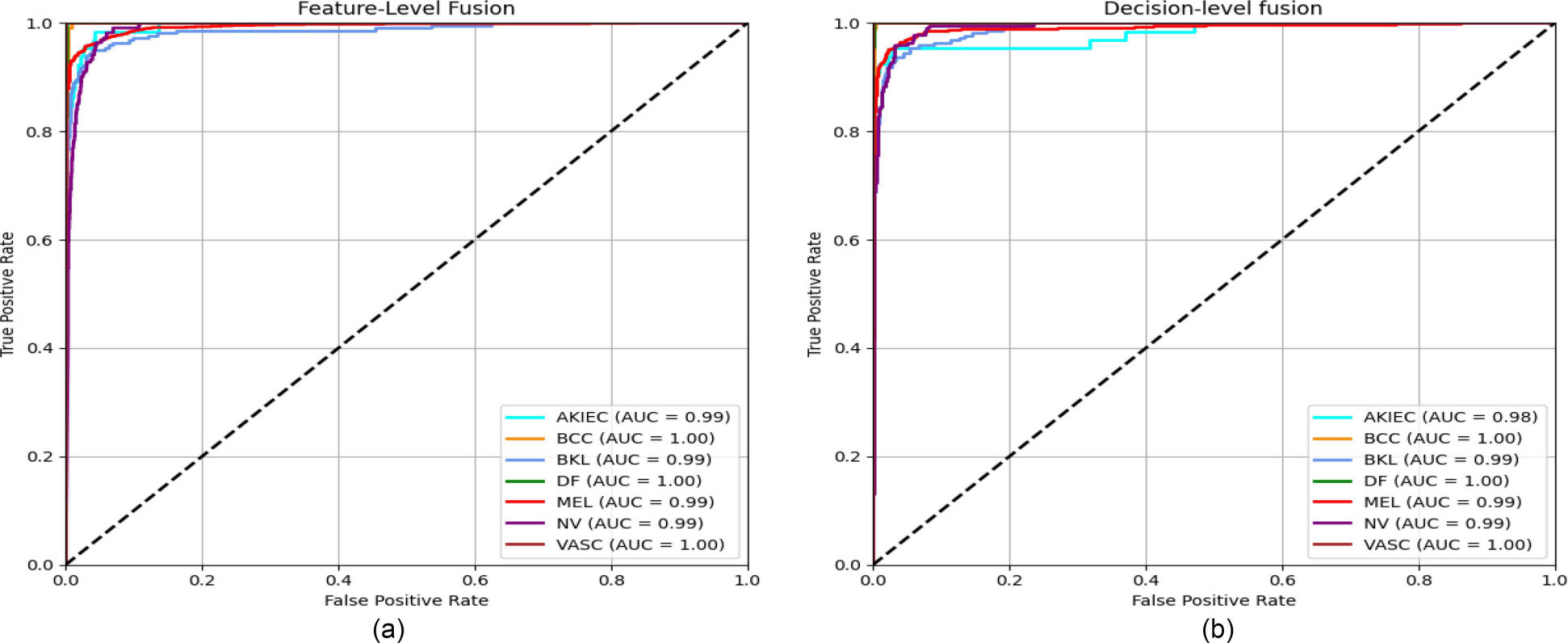
ROC curves for the FLF and DLF approaches on the HAM10000 dataset, with corresponding AUC values included in the legend. **(a)** Feature-level fusion. **(b)** Decision-level fusion.

The accuracy, precision, recall, and F1-score on the HAM10000 dataset are presented in Table 3 and Supplementary Figure S1, along with the ROC curves and corresponding AUC values in Figure 7. The confusion matrices of our proposed approaches are shown in Figure 6. Compared with existing well-established models including the pre-trained CNN-based models ResNet50 (75), EfficientNetB4 (77), EfficinetNetB1 (80) and Xception (97), custom CNN (78, 79, 82), and studies with attention-based or combined with a CNN-based approach (49, 76, 81), our proposed end-to-end FLF achieves 92.7%, 93.5%, 92.6%, and 92.8% accuracy, precision, recall, and F1-score. On the other hand, Our DLF achieves 96.1%, 96.2%, 96.1%, 96.1% accuracy, precision, recall, and F1-score. Notably, we also compared our approach with the recent CNN along with ViT-based hybrid method proposed in (83), which achieved 95.0% accuracy, 94.7% precision, 92.1% recall, and 93.3% F1-score.

**Table 3:** Comparison of the proposed framework with existing methods applied to the HAM10000 dataset.

Reference	Method	Accuracy	Precision	Recall	F1-score
Liu et al. (74)	CNN	92.5	–	71.5	60.7
Al et al. (75)	ResNet50	89.3	–	81.0	81.3
Nie et al. (49)	CNN+Attention	89.5	89.6	89.5	89.1
Cai et al. (76)	Attention	93.9	–	90.1	90.1
Ali et al. (77)	EfficientNetB4	87.9	88.0	88.0	87.0
Shetty et al. (78)	CNN	95.2	88.0	85.0	86.0
Wu et al. (79)	ResNet50	*95.8*	*96.0*	*96.0*	*96.0*
Tajerian et al. (80)	EfficientNetB1	84.3	73.4	67.4	70.0
You et al. (81)	Attention+CNN	80.4	–	–	–
Wei et al. (82)	DenseNet+ConvNeXt	90.9	83.8	83.8	83.5
Mustafa et al. (73)	ResUNet+AlexNet	92.0	–	–	–
Pacal et al. (83)	CNN + ViT	95.0	94.7	92.1	93.3
Our	FLF (DenseNet201+VGG19+ViT)	92.7	93.5	92.6	92.8
Our	DLF (DenseNet201+VGG19+ViT)	**96.1**	**96.2**	**96.1**	**96.1**

Bold and italic values indicate the best and second-best benchmarks, respectively.

Furthermore, our proposed DLF framework surpasses the best-performing existing benchmarks by 0.3%, 0.2%, 0.1%, and 0.1% in terms of accuracy, precision, recall, and F1-score, respectively. To further validate the robustness of our method, we performed a bootstrap analysis with 1,000 iterations to compute 95% confidence intervals (CIs) for the key performance metrics. The results are summarized in Table 4. Our DLF achieved an accuracy of 96.1% [95% CI: 95.2%, 96.9%], while the second-best method achieved an accuracy of 95.8%. Notably, the lower bound of our method’s CI (95.2%) is close to the mean accuracy of the second-best method, indicating a consistent—though modest—improvement. Similarly, the precision, recall, and F1-score exhibit tight confidence intervals, reflecting stable and reliable performance across multiple resamples. These findings statistically reinforce that our method offers a robust and consistent improvement over the existing benchmarks, with reduced variability in performance.

**Table 4:** Bootstrap results with 95% confidence intervals (CI) for the decision-level fusion (DLF) method on the HAM10000 dataset for 1,000 iterations.

Metric	Mean	CI lower	CI upper
Accuracy [%]	96.1	95.2	96.9
Precision [%]	96.2	95.4	97.0
Recall [%]	96.1	95.2	96.9
F1-score [%]	96.1	95.2	96.9

#### 5.2.3 Evaluation on the ISIC 2018 dataset

**Figure 8:**
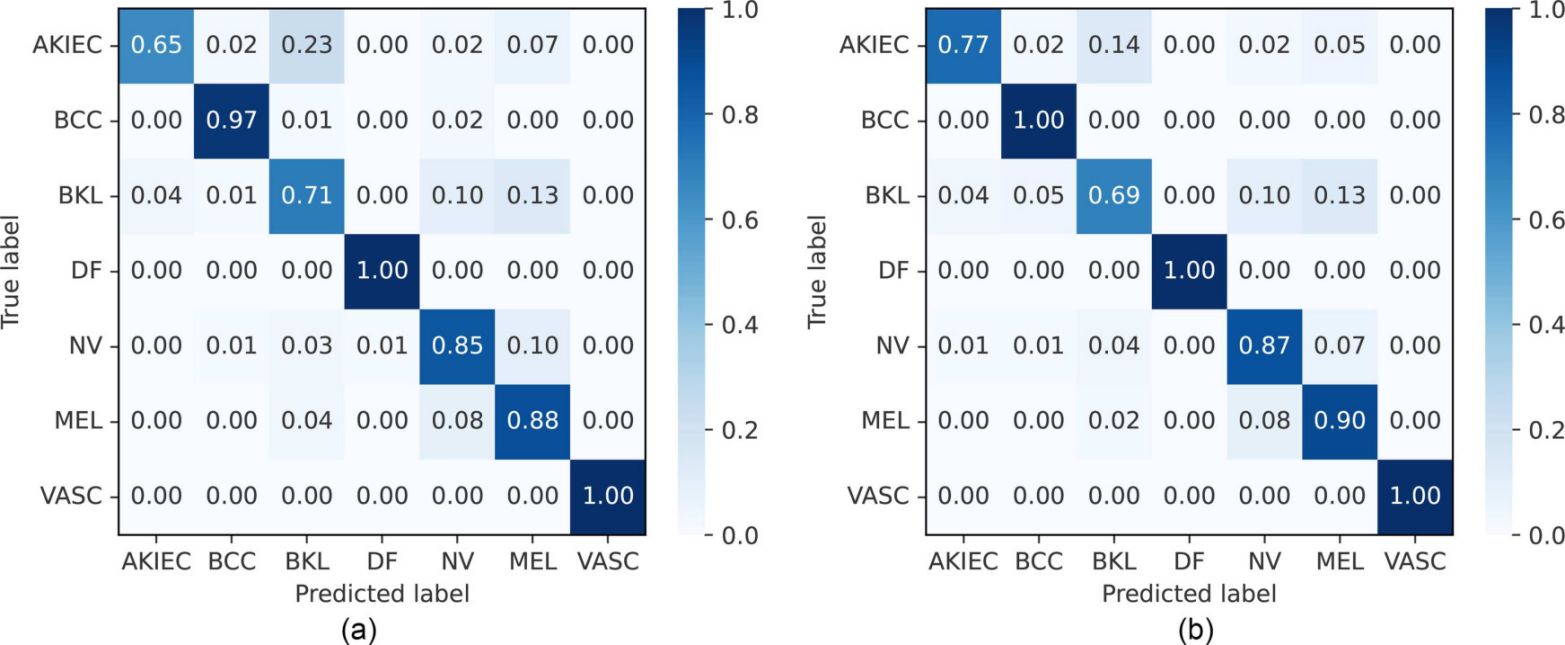
Normalized confusion matrices of FLF and DLF on the ISIC 2018 challenge test dataset. AKIEC, actinic keratosis; BCC, basal cell carcinoma; BKL, benign keratosis; DF, dermatofibroma; NV, melanocytic nevi; MEL, melanoma; VASC, vascular lesions. **(a)** Feature-level fusion. **(b)** Decision-level fusion.

The balanced accuracy, precision, recall, and F1-score on the ISIC 2018 dataset are presented in Table 5 and Supplementary Figure S1, along with the confusion matrix of our approaches, which is shown in Figure 8. Compared with CNN-based models (84, 87–90), our proposed FLF approach has achieved 86.7%, 97.0%, 84.6%, and 85.2%, respectively, for balanced accuracy, specificity, recall, and F1-score while 89.0%, 97.3%, 86.1%, and 86.4% for DLF, respectively. This implies the supremacy of our proposed approaches, where the DLF approach achieves 0.5% higher balanced accuracy than the best-performing existing benchmarks. Our observations demonstrate that decision-level fusion (DLF) achieves superior benchmark performance among the evaluated methods.

**Table 5:** Comparison of the proposed framework with existing methods applied to the ISIC 2018 dataset.

Reference	Method	B. Acc.	Specificity	Recall	F1-score
Nozdryn et al. (84)	CNN	*88.5*	**98.6**	83.3	–
Gessert et al. (85)	DenseNet+ResNeXt+SENets	85.6	*98.4*	80.9	–
Zhuang et al. (86)	SENet+PNASNet	84.5	98.0	80.4	–
Mahbod et al. (87)	EfficientNetB0+EfficientNetB1+SeReNeXt50	86.2	–	–	–
Shen et al. (88)	EfficientNetB0	85.3	97.3	–	–
Barata et al. (89)	CNN	79.1	–	–	–
Tsai et al. (90)	CNN	82.1	–	–	–
Our	FLF(DenseNet201+VGG19+ViT)	86.7	97.0	*84.6*	*85.2*
Our	DLF(DenseNet201+VGG19+ViT)	**89.0**	97.3	**86.1**	**86.4**

Bold and italic values indicate the best and second-best benchmarks, respectively. Here, B.Acc. indicates the balance accuracy.

#### 5.2.4 Evaluation on the ISIC 2019 dataset

**Figure 9:**
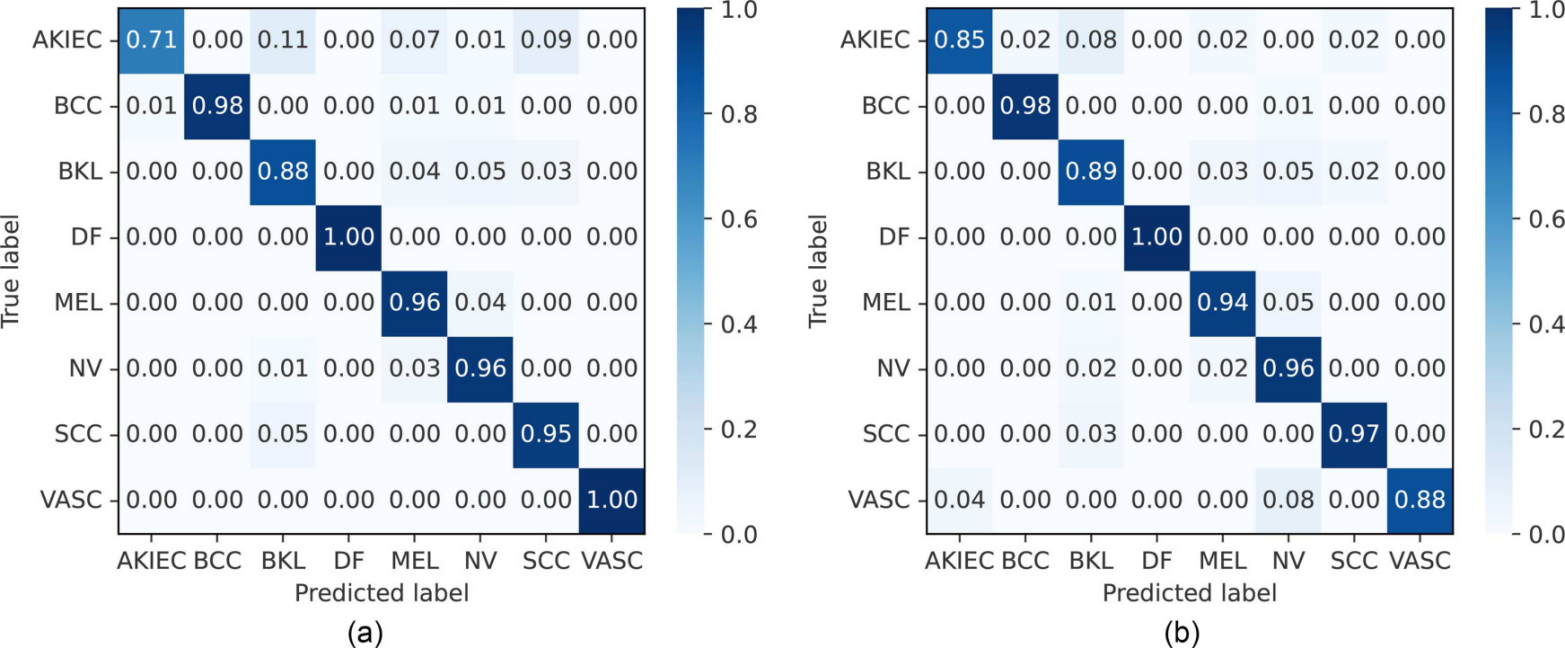
Normalized confusion matrices of FLF and DLF on the ISIC 2019 dataset. AKIEC, actinic keratosis; BCC, basal cell carcinoma; BKL, benign keratosis; DF, dermatofibroma; NV, melanocytic nevi; MEL, melanoma; SCC, squamous cell carcinoma; VASC, vascular lesions. **(a)** Feature-level fusion. **(b)** Decision-level fusion.

The accuracy, precision, recall, and F1-score on the ISIC 2019 dataset are presented in Table 6 and Supplementary Figure S1, along with the confusion matrix of our approaches, which is shown in Figure 9. Comparing the CNN-based model with an SVM (91), GoogleNet, and DarkNet (96), EfficientNets, SENet, and ResNeXt (92), and the single end-to-end CNN-based model MobileNetV2 (95), our proposed FLF framework achieves 94.5%, 94.7%, 94.4%, and 94.4% accuracy, precision, recall, and F1-score, respectively, while 95.0%, 94.9%, 94.8%, and 94.8% for DLF, respectively. This implies that our proposed end-to-end FLF ensemble framework achieves comparable performance while DLF slightly improves over the existing best-performing benchmark. For example, DLF surpasses by 2.9% and 1.8%, respectively, for the precision and recall from the existing best-performing benchmark [i.e., the approach in (94)] while by 2.7%, and 1.4% for FLF.

**Table 6:** Comparison of the proposed framework with existing methods applied to the ISIC 2019 dataset.

Reference	Method	Accuracy	Precision	Recall	F1-score
Kassem et al. (91)	GoogleNet+SVM	*94.9*	80.4	79.8	–
Gessert et al. (92)	EfficientNets+SENet+ResNeXt	63.0	–	73.0	–
Bhardwa et al. (93)	CNN+SVM	86.0	80.0	60.0	–
Jain et al. (94)	DNN	**95.0**	92.0	93.0	–
Wang et al. (95)	MobileNetV2	84.6	–	–	–
Abdelhafeez et al. (96)	GoogleNet+DarkNet+SVM	85.7	84.0	76.1	–
Mustafa et al. (73)	ResUNet+AlexNet	93.4	–	–	–
Pacal et al. (83)	CNN + ViT	92.5	90.4	87.7	88.9
Our	FLF(DenseNet201+VGG19+ViT)	94.5	*94.7*	*94.4*	*94.4*
Our	DLF(DenseNet201+VGG19+ViT)	**95.0**	**94.9**	**94.8**	**94.8**

Bold and italic values indicate the best and second-best benchmarks, respectively.

## 6 Discussion

Our proposed framework leverages an ensemble approach that integrates two convolutional neural networks (CNN)-based architectures: a modified DenseNet201 and a VGG19. Additionally, it incorporates an attention-based vision transformer model, ViT. To address data scarcity, we employed a pre-training strategy utilizing a generative adversarial network (GAN) (65) for generating image samples artificially. We also added samples from other ISIC archives. Moreover, we added other online data augmentation techniques during training. Here, we conduct a comparative analysis of the performance achieved by our proposed framework against various baselines (i.e., base models w/o Data Augmentation (DA), DA with GAN (DA_GAN), and ISIC archives (DA_Archive)). Additionally, we also compared each of the base models. Similarly, we further delve into an in-depth performance analysis of the FLF and DLF for the final classification. In this section, for ablation studies of our proposed framework, we selected the small-scale and large-scale datasets PH2 and ISIC 2019 and adhered to the identical protocol outlined in Section 4.1 for these analyses.

### 6.1 Impact of data augmentation

We illustrate the impact of data augmentation for each of the base models, modified VGG19, DenseNet201 and ViT along with the proposed frameworks in Table 7. We can observe that the accuracy is improved by a large margin for a small-scale dataset (i.e., PH2 dataset) when employing the augmentation using GAN as well as ISIC archives. As shown in Table 7, the accuracy is improved from 11.6% to 22.8% when we increase the training sample size using the deep generative approach. Moreover, improvement continues when the training data volume is again increased by adding samples from ISIC archives. Overall, we can see that the accuracy is improved from 15.2% to 25.4% when we augment the training dataset using a generative approach and add samples from ISIC archives. We think that this large margin accuracy improvement for the small-scale dataset PH2 when augmenting the training dataset because large-scale training datasets are essential for the DL-based approach for effective training and generalization.

**Table 7:** Result of each base model before and after data augmentation.

Dataset	Method	DA_GAN	DA_Archive	Accuracy	Precision	Recall	F1-score
PH2	VGG19	✗	✗	70.0	71.6	70.0	68.9
		✓	✗	88.9	89.0	88.9	88.9
		✓	✓	93.4	93.5	93.4	93.4
	DenseNet201	✗	✗	72.5	74.0	72.5	72.6
		✓	✗	95.3	95.3	95.3	95.3
		✓	✓	97.9	97.9	97.9	97.9
	ViT	✗	✗	80.0	83.6	80.0	79.8
		✓	✗	95.2	95.4	95.2	95.2
		✓	✓	98.8	98.8	98.8	98.8
	Our FLF	✗	✗	82.5	84.1	82.5	82.6
		✓	✗	96.9	95.7	95.5	95.5
		✓	✓	99.3	99.3	99.3	99.3
	Our DLF	✗	✗	75.0	75.2	75.0	74.9
		✓	✗	95.4	95.4	95.4	95.4
		✓	✓	98.2	98.2	98.2	98.2
ISIC 2019	VGG19	✗	✗	82.0	82.0	82.0	82.0
		✓	✗	85.1	85.0	85.1	85.0
		✓	✓	86.5	87.4	86.5	86.7
	DenseNet201	✗	✗	90.0	90.0	90.0	90.0
		✓	✗	90.8	90.7	90.8	90.7
		✓	✓	93.0	93.3	93.0	93.1
	ViT	✗	✗	91.6	91.5	91.6	91.5
		✓	✗	92.4	92.3	92.4	92.3
		✓	✓	94.2	94.3	94.2	94.1
	Our FLF	✗	✗	91.7	91.6	91.6	91.5
		✓	✗	92.4	92.3	92.4	92.3
		✓	✓	94.5	94.7	94.4	94.4
	Our DLF	✗	✗	91.9	91.8	91.8	91.7
		✓	✗	92.6	92.5	92.6	92.5
		✓	✓	95.0	94.9	94.8	94.8

In contrast, for the large-scale dataset ISIC 2019, we observed marginally improved accuracy when employing data augmentation techniques. For instance, the accuracy is improved from 0.7% to 0.8% when the training sample is augmented by a generative approach GAN. A similar tendency we observed when we added samples from ISIC archives. In general, the accuracy is improved by around 3.0% when the training data is augmented using GAN and ISIC archives. This modest improvement can be attributed to the inherent characteristics of the ISIC 2019 dataset. As a large-scale dataset encompassing 22,797 samples, it already possesses a high degree of diversity and quantity, providing a sufficient foundation for robust model training.

### 6.2 Impact of individuals module

We evaluated each of the base models considered in our framework separately: The modified VGG19, DenseNet201 and ViT. We can observe that the vision transformer-based ViT model works better than the CNN-based model. For example, the accuracy of the ViT model with data augmentation is 98.8% on the small-scale dataset PH2 while 97.9%/93.4% for the DenseNet201/VGG19. Regarding the large-scale dataset ISIC 2019, we observe a similar tendency that the ViT model works better than the CNN-based approach DenseNet201 and VGG19. We think that ViT-based models work better because they capture global and local contexts more effectively and learn complex relationships without relying on fixed receptive fields. Furthermore, unlike the CNN-based approach, ViT leverages self-attention mechanisms to consider interactions between image patches, enabling them to better understand long-range dependencies crucial for tasks like detection and classification.

Regarding the CNN-based approaches of the modified DenseNet201 and VGG19, we can observe that DenseNet201 works better than VGG19. For example, DenseNet201 obtained accuracy at 97.9% on the small-scale dataset PH2 while 93.4% on the large-scale dataset ISIC 2019. This indicates that it surpassed 4.5% and 6.5% from the VGG19, respectively, for the PH2 and ISIC 2019 datasets. We think that it may be the cause of reason, such as VGG19 is a relatively straightforward network where each layer feeds into the next. At the same time, DenseNet201 incorporates dense connections, where each layer receives additional inputs from all preceding layers and passes its feature maps to all subsequent layers. This characteristic allows for feature reuse throughout the network, consequently enhancing model performance and mitigating the risks of overfitting and vanishing gradients.

**Figure 10:**
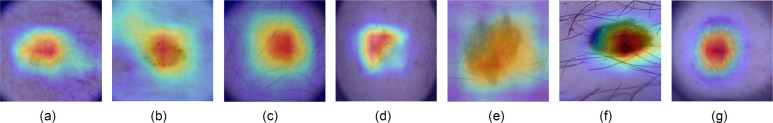
Grad-CAM visualizations of different skin lesion classes from the HAM10000 dataset, where AKIEC, actinic keratosis; BCC, basal cell carcinoma; BKL, benign keratosis; DF, dermatofibroma; NV, melanocytic nevi; MEL, Melanoma; VASC, vascular lesions. **(a)** AKIEC. **(b)** BCC. **(c)** BKL. **(d)** DF. **(e)** NV. **(f)** MEL. **(g)** VASC.

To further assess the interpretability and clinical relevance of the models, we generated Grad-CAM (98) visualizations using the DenseNet201 architecture. These heatmaps highlight the image regions that most strongly contributed to each prediction, showing that the model predominantly focuses on lesion areas rather than irrelevant background. An example Grad-CAM activation map is presented in Figure 10, demonstrating the alignment between the model’s attention and dermatological diagnostic regions.

### 6.3 Impact of the attention mechanism

Our framework employs the MHSA mechanism of ViT (44). To assess the impact of using an attention-based model, we compare the performance of ViT against CNN-based models (i.e., DenseNet201 and VGG19). ViT consistently outperforms CNN models, achieving an accuracy of 98.8% on PH2 compared to 97.9% and 93.4% for DenseNet201 and VGG19, respectively. A similar trend is observed on ISIC 2019. These results confirm that the inclusion of MHSA into the ViT improves accuracy and the ViT’s ability to capture long-range dependencies and global contextual features.

### 6.4 Comparison with feature and decision-level fusion

For the final classification stage of our proposed ensemble model, we employed a fusion strategy that leverages both feature-level fusion (FLF) and decision-level fusion (DLF). The performance acheived by this framework is presented in Tables 2, 3, 5, 6. Supplementary Figure S2 includes the classification report figures for FLF and DLF models, based on the HAM10000, ISIC 2018, and ISIC 2019 datasets. We can observe that, DLF exhibits marginally superior performance compared to the end-to-end FLF model. For example, the DLF surpasses the accuracy by 2.3% from FLF for the ISIC 2018 dataset and 0.5% for the ISIC 2019 dataset. This may cause robust training for individual models and merge the individual decision from the respective classifier. However, the decision-level fusion (DLF) necessitates a longer processing time to arrive at the final classification result, and it is not an end-to-end process.

### 6.5 Comparison of different decision-level fusion techniques

We performed various fusion strategies for decision-level fusion, specifically employing averaging voting, weighted averaging voting, and majority voting (99). Averaging voting (AVG) refers to taking the mean of the prediction scores from base classifiers to make the final decision, while weighted averaging voting (WAVG) applies different weights to these scores. For the weighted average case, we empirically assigned weights of 0.4, 0.3, and 0.3 to the prediction scores of ViT, DenseNet201, and VGG19, respectively. These weightings were determined through a sensitivity analysis, which revealed that the selected values provide the best balance between model performance across the HAM10000, ISIC 2018, and ISIC 2019 datasets. The majority voting (MJ) technique, as described in Section 3.3, involves selecting the class that appears most frequently among the predictions of the base classifiers. The results are presented in Table 8. Our observations show that the MJ technique achieves superior accuracy, while AVG and WAVG perform almost equally. This superiority of MJ can be attributed to its core principle of aggregating predictions and selecting the most frequent class, which reduces the impact of outliers or misclassifications from individual base models.

**Table 8:** Performance evaluation of our proposed ensemble framework for decision-level fusion using different fusion techniques [average (AVG), weighted average (WAVG), and majority voting (MJ)].

Dataset	Method	Accuracy	Precision	Recall	F1-score
PH2	AVG	97.9	98.0	97.9	97.9
	WAVG	98.1	98.1	98.1	98.1
	MJ	98.2	98.2	98.2	98.1
ISIC 2019	AVG	93.8	93.9	93.8	93.8
	WAVG	93.9	94.0	93.9	93.8
	MJ	95.0	94.9	94.8	94.8

### 6.6 Cross-dataset evaluation

To assess the generalizability and robustness of the proposed approach, we conducted a cross-dataset evaluation by training the models on the PH2 dataset and testing them on the Derm7pt test dataset (100). This setup simulates a real-world scenario in which a model is trained on a small-scale dataset and applied to an independent large-scale dataset with potentially different data distributions. The Derm7pt dataset includes five general disease classes: melanoma, nevus, seborrheic keratosis, basal cell carcinoma, and miscellaneous. In our experimental setting, we focused on the two disease classes common to the PH2 dataset (i.e., nevus and melanoma). For this purpose, we merged common nevus (CN) and atypical nevus (AN) into a single nevus class. The experimental results are shown in Table 9 for each of the base models: ViT, DenseNet201, and VGG19, as well as our proposed FLF and DLF approaches. We can observe that DenseNet201 achieved the highest accuracy (80.6%) and F1-score (79.6%) among the individual models. Compared to all base models, the FLF ensemble yielded the best overall performance, with an accuracy of 82.1%, precision of 82.4%, recall of 82.1%, and F1-score of 80.8%. The DLF approach also outperformed the individual models, achieving 81.3% accuracy and an F1-score of 79.4%. These results demonstrate that the proposed fusion frameworks improve generalization and robustness for cross-dataset evaluation.

**Table 9:** Cross-dataset evaluation results: Models trained on the PH2 dataset and tested on the Derm7pt dataset.

Method	Accuracy	Precision	Recall	F1-score
VGG19	79.6	80.5	79.6	77.3
DenseNet201	80.6	80.0	80.6	79.6
ViT	78.4	79.0	78.4	75.8
Our FLF	**82.1**	**82.4**	**82.1**	**80.8**
Our DLF	*81.3*	*81.9*	*81.2*	*79.4*

Bold and italic values indicate the best and second-best benchmarks, respectively.

## 7 Conclusion

Skin disease is one of the most prevalent and potentially life-threatening diseases that has affected people all over the world. Early detection and treatment are crucial for improving patient outcomes. However, the subjective nature of the healthcare providers’ approach to early diagnosis can be both costly and unpredictable, potentially leading to variable results in patient care. In this paper, we proposed a deep learning-based ensemble model, including CNN-based base models and an attention-based vision transformer network for diagnosing skin diseases. The proposed framework considers the feature-level fusion (FLF) that is extracted from each of the base models and merges them through pointwise addition in a separated layer along with a final classification layer with Softmax. We employed the decision-level fusion (DLF) by employing the majority voting for each classification result.

To evaluate the proposed framework, we employed four publicly available datasets encompassing ten distinct skin diseases: Actinic keratosis, Basal cell carcinoma, Benign keratosis, Dermatofibroma, Melanocytic nevi, Melanoma, Squamous cell carcinoma, Common nevi, Atypical nevi, and Vascular lesions. We assessed performance using standard metrics: accuracy, precision, recall, and F1-score. Our results demonstrate that the proposed FLF and DLF outperform existing methods. The experimental evaluation shows the majority voting techniques’ effectiveness over other ensemble techniques like Averaging and Weighted Averaging. Furthermore, we conducted a comprehensive analysis of each base model within the proposed framework, revealing a significant accuracy improvement attributable to the framework itself. Additionally, we employed a variety of online and offline data augmentation methods to expand the training dataset, mitigate overfitting, and enhance model generalizability. It is evident from our findings that data augmentation significantly enhances accuracy. Despite these promising results, the proposed approach has certain limitations. Particularly, the architecture of the proposed ensemble model requires the concurrent training and inference of two CNN-based models and an attention-based Vision Transformer (ViT) model, leading to increased training time and demanding significant computational resources. Therefore, this may affect practical challenges in resource-constrained real-time clinical settings. Future work could explore a more lightweight ensemble model to mitigate these constraints. Additionally, future architectures could best investigate a potential closed loop between data generation and data analysis to avoid the explicit generation and training of data.

Since our model uses the canonical MHSA without modification, an additional ablation comparing alternative attention mechanisms is outside the scope of this study but is suggested as future work.

## Data Availability

The raw data supporting the conclusions of this article will be made available by the authors, without undue reservation.

## Footnotes

^1^ https://challenge.isic-archive.com/data/#2018

^2^ https://challenge.isic-archive.com/data/#2019

^3^ https://www.isic-archive.com/
