# Supplementary material for: Skin disease diagnosis using decision and feature level fusion of deep features
Source: Front Digit Health. 2025 Oct 17;7:1478688. doi: 10.3389/fdgth.2025.1478688 (PMC12575228; doi:10.3389/fdgth.2025.1478688)
Supplement: Supplementary file 1 [file Datasheet1.pdf]

Skin Disease Diagnosis using Decision and Feature Level Fusion of Deep Features

1 SUPPLEMENTARY MATERIAL

1.1 Supplementary Appendix S1

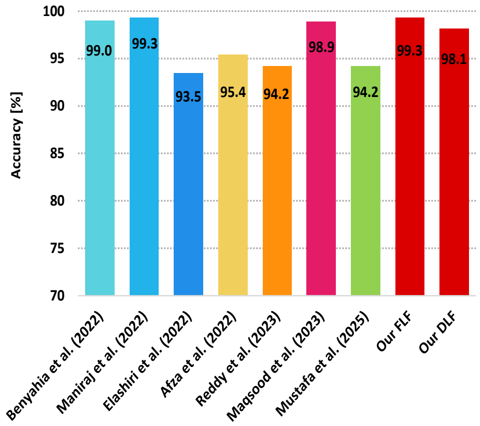

Figure 1a. PH2

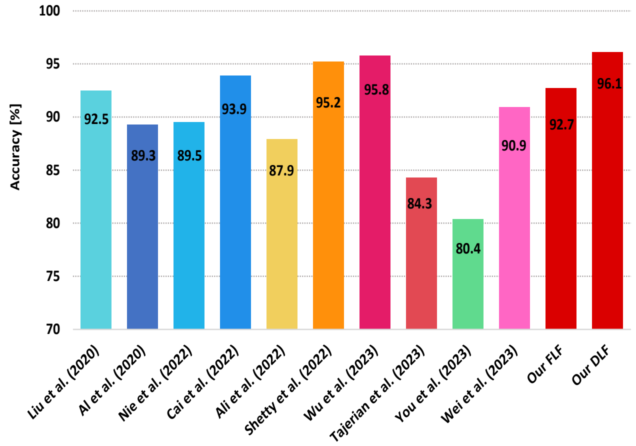

Figure 1b. HAM10000

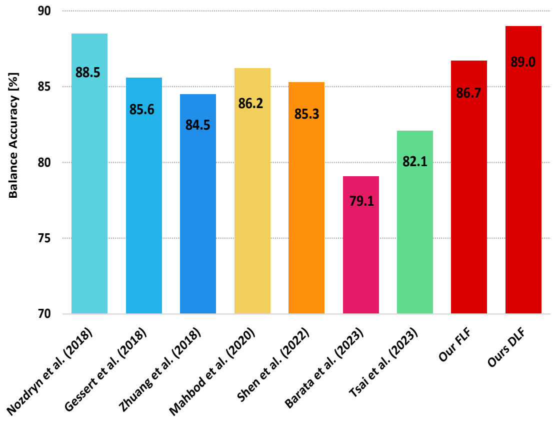

Figure 1c. ISIC 2018

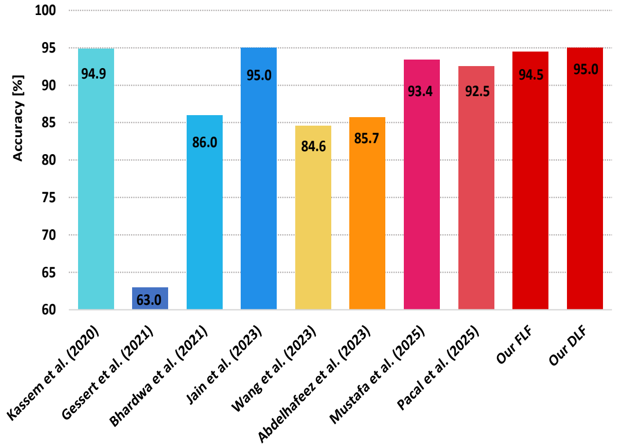

Figure 1d. ISIC 2019

Figure 1. Comparison of our method with state-of-the-art approaches. (a) PH2 dataset (b) HAM10000 dataset (c) ISIC 2018 dataset (d) ISIC 2019 dataset. Note that the scales used differ among the graphs.

1.2 Supplementary Appendix S2

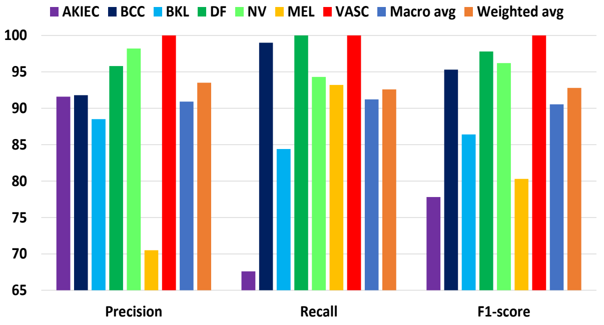

Figure 2a. FLF of HAM10000

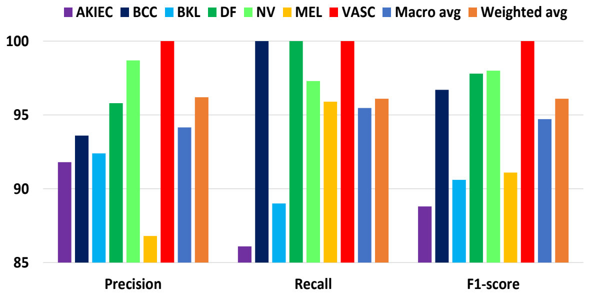

Figure 2b. DLF of HAM10000

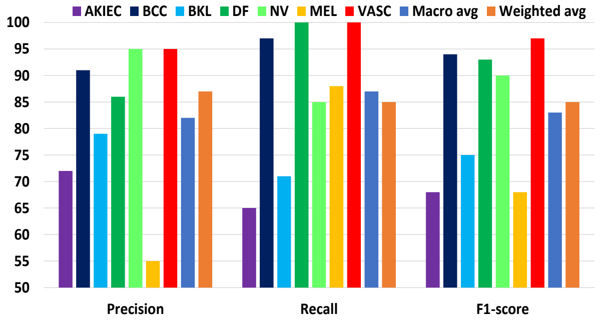

Figure 2c. FLF of ISIC 2018

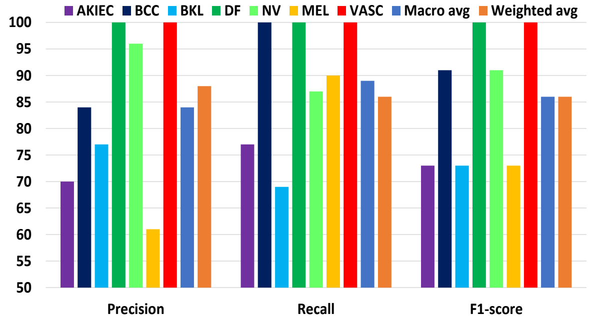

Figure 2d. DLF of ISIC 2018

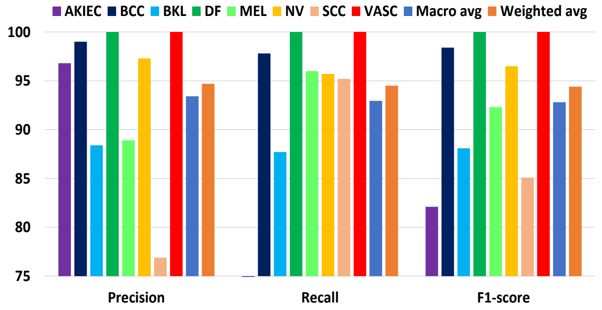

Figure 2e. FLF of ISIC 2019

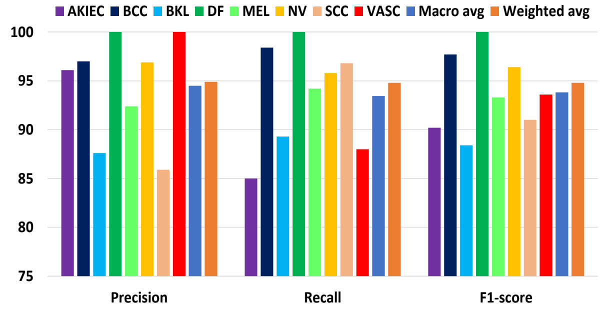

Figure 2f. DLF of ISIC 2019

Figure 2. Presents the classification report of feature-level fusion (FLF) (left) and decision-level fusion (DLF) (right) on HAM10000, ISIC 2018, and ISIC 2019 datasets.
